# Testing the accuracy of the DRNNAGE software for age estimation in a modern Greek sample

**DOI:** 10.1007/s00414-023-03129-4

**Published:** 2023-11-25

**Authors:** Leuteris Rizos, Nefeli Garoufi, Eustratios Valakos, Efthymia Nikita, Maria-Eleni Chovalopoulou

**Affiliations:** 1 Department of Animal and Human Physiology, Faculty of Biology, School of Sciences, National and Kapodistrian University of Athens, Panepistimiopolis, 157 01 Athens, GR Greece; 2 Science and Technology in Archaeology and Culture Research Center, The Cyprus Institute, Aglantzia, 2121 Nicosia, Cyprus

**Keywords:** Age-at-death estimation, DRNNAGE software, Greek sample, Validation

## Abstract

Estimation of age-at-death from human skeletal remains is fundamental in forensic anthropology as part of the construction of the biological profile of the individual under study. At the same time, skeletal age-at-death estimation in adults is problematic due to the disparity between chronological and biological age, the important inter-individual variability at the rate of skeletal aging, and inherent biases in the available methodologies (e.g., age mimicry). A recent paper proposed a method for skeletal age-at-death estimation based on multiple anatomical traits and machine learning. A software was also created, DRNNAGE, for the easy implementation of this method. The authors of that study supported that their methods have very high repeatability and reproducibility, and the mean absolute error of the age estimation was ~6 years across the entire adult age span, which is particularly high and promising. This paper tests the proposed methodology on a modern documented Greek sample of 219 adult individuals from the Athens Collection, with age-at-death from 19 to 99 years old. The sample was split into males and females as well as into individuals under and over 50 years old. We also divided the sample in 10-year intervals. First, intra- and inter-observer error was estimated in order to assess repeatability and reproducibility of the variables employed for age-at-death estimation. Then, the validity (correct classification performance) of DRNNAGE for each anatomical region individually, as well as all combined, was evaluated on each demographic separately and on the pooled sample. According to the results, some of the variables showed very low repeatability and reproducibility, thus their use should be cautious. The DRNNAGE software showed overall highly accurate age-at-death estimates for individuals older than 50 years, but poor on younger adults, with only exception the cranial sutures, which performed surprisingly well for all age groups. Overall, these results support the importance of cross-validation and the use of population-specific methods in forensic anthropology.

**Supplementary Information:**

The online version contains supplementary material available at 10.1007/s00414-023-03129-4.

## Introduction

Estimation of skeletal age-at-death is fundamental in forensic anthropology as it is among the key aspects used in the construction of the biological profile of the individual under study. Several methods focused on the analysis of skeletal and dental structures have been proposed for biological age-at-death estimation, that is, age estimation from skeletal remains [1]. For juveniles, such methods are overall highly accurate because they are based on developmental processes [2]. In contrast, for adult individuals, age-at-death estimation is based mainly on skeletal degenerative changes, which may be affected by the individual’s activity levels, diseases, dietary patterns, and other factors. Therefore, they exhibit inter-individual and inter-population variation at their timing and rate of expression [3–8]. This issue is exacerbated in older individuals since during an individual’s lifetime, biological age tends to increasingly diverge from chronological age, that is, the amount of time that has elapsed since an individual’s birth [9].

An additional important bias is the so-called age mimicry, that is, when using regression models to predict chronological age, the age estimates, to some degree, reflect the age structure of the reference sample [10]. Attempts to overcome age mimicry have employed Transition Analysis and Bayesian statistics, which calculate the probability of an individual having made the transition from one skeletal change stage to the next at each age [11]. However, Bayesian analysis requires prior knowledge of the distribution of ages-at-death in the population, which is often not available. At the same time, several studies have found that Bayesian statistics and Transition Analysis do not improve age predictions compared to traditional methods [12, 13].

An alternative approach has been the combined use of multiple age-related traits across the skeleton [14], an approach also supported by the scholars working on the revised version of Transition Analysis [11]. In this direction, a recent paper proposed the study of several age markers across the human skeleton (up to 64 skeletal traits from seven anatomical regions), using novel recording criteria and reaching age-at-death predictions via machine learning based on deep randomized neural networks [14]. To facilitate the implementation of this method, the authors developed an open access software, DRNNAGE. According to the team who developed this initiative, the application of this method on a Portuguese assemblage resulted in a mean absolute error of the age estimation of ~6 years across the entire adult age span. These results are impressive and, if confirmed in other test assemblages, they are very promising in forensic anthropology.

Indeed, any forensic anthropological method needs to be validated by testing its accuracy on diverse assemblages. Therefore, this work aims to test the accuracy of the DRNNAGE software in age-at-death estimation using a documented modern Greek sample.

## Materials and methods

**Fig. 1:**
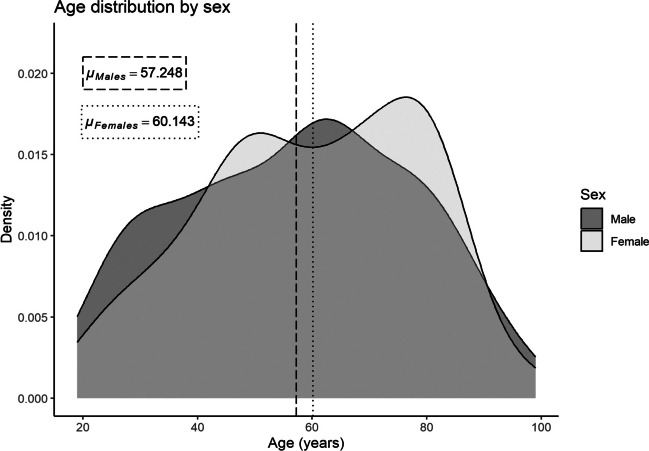
Age-at-death distribution of the sample for the male and female subgroups. The mean age-at-death for each group is represented with a dashed and dotted line for males and females accordingly

The present study uses 219 adult individuals (121 males and 98 females) from the Athens Collection, housed in the Department of Animal and Human Physiology of the National and Kapodistrian University of Athens. Information on the sex, age-at-death, occupation, cause of death, and place of birth of each individual in the collection is derived from death records [13, 15]. The year of birth for all individuals ranges between 1879 and 1965, while their age-at-death ranges from 19 to 99 years old. The age distribution of the assemblage per sex is presented in Fig. 1, while Table 1 shows the age and sex distribution of the skeletons in relation to the different anatomical regions recorded. The individuals were selected so as to maximize sample sizes per sex and age group, while maintaining a balanced representation of all demographics. Nonetheless, as is common in modern documented collections, there is an over-representation of older (> 50 years) individuals. To avoid biases in the results, analyses were performed for the pooled age and sex group sample but also separately for males-females and per age group (younger or older than 50 years). Furthermore, for better evaluation of the DRNNAGE performance, analyses were performed in a pooled sex sample on a decade-based segmentation. Any individuals with evidence of pathological deformation that could have affected the recording of the age-related changes required for DRNNAGE were excluded from the assemblage.

**Table 1:** Sample distribution per anatomical region for each examined scenario

	Anatomical regions								
	All regions	Cranial sutures	Vertebrae	Upper limb	Lower limb	Clavicle and 1st rib	Pubic symphysis	Sacroiliac joint	Acetabulum
Pooled	219	217	216	219	219	214	183	214	217
Female	98	98	97	98	98	95	74	97	97
Male	121	119	119	121	121	119	109	117	120
Individuals < 50 yo	82	80	81	82	82	78	66	82	82
Individuals > 50 yo	137	137	135	137	137	136	117	132	137
Female < 50 yo	34	34	34	34	34	32	26	34	33
Female > 50 yo	64	64	63	64	64	63	48	63	64
Male < 50 yo	48	46	47	48	48	46	40	48	47
Male > 50 yo	73	73	72	73	73	73	69	69	73
Individuals 19–29 yo	23	23	23	23	23	22	12	23	23
Individuals 30–39 yo	21	19	20	21	21	20	14	21	20
Individuals 40–49 yo	33	33	33	33	33	31	31	33	32
Individuals 50–59 yo	32	32	32	32	32	32	28	32	32
Individuals 60–69 yo	39	39	37	39	39	39	34	37	39
Individuals 70–79 yo	34	34	34	34	34	33	27	34	34
Individuals 80–89 yo	31	31	31	31	31	31	27	28	31
Individuals > 90 yo	6	6	6	6	6	6	5	6	6

The DRNNAGE software can be accessed at the webpage https://osteomics.com/DRNNAGE/. It utilizes up to 64 skeletal traits (Table S1), which encode both developmental and degenerative aspects from seven anatomical regions: cranial sutures, vertebrae, upper and lower limbs, pubic symphysis, sacroiliac joint, acetabulum, clavicle, and first rib. These seven regions may be used individually for age estimation as well as in combination. The DRNNAGE software can handle missing values in the input dataset, which is particularly important given the damaged and partial preservation of many skeletons due to taphonomic factors. The researcher can choose one of the four available network algorithms (randomized network, ensembled randomized network, ensemble autoencoder—U, and ensemble autoencoder—S) to estimate the age-at-death of an unknown specimen.

The classification performance of DRNNAGE for each anatomical region individually and the combination of all was evaluated in this paper utilizing all four available network algorithms on each demographic group of the Athens Collection separately and on the pooled sample. All tests were ran using the software’s default settings regarding the parameters of the neural networks. 1 In the present article, the tables present the results of the ensembled randomized network (ERN) for age-at-death estimation. The results of the other three network algorithms are provided in the Supplementary Material.

All skeletal traits were scored by the first author (RE), and input to the DRNNAGE software for age estimation. Individuals were classified as correctly aged when their documented age-at-death lay in the age range estimated by the DRNNAGE software. Furthermore, the reliability of the estimates was tested by measures of bias and inaccuracy, where bias expresses the mean over- or under-prediction of an individual’s chronological age and is estimated as Σ(estimated age − actual age)/*n*, and inaccuracy expresses the average absolute error of age estimation and is estimated as Σ|estimated age − actual age|/*n*. In these estimates, the actual age was the documented age-at-death of each individual and the estimated age was obtained from DRNNAGE.

To assess intra- and inter-observer error, all traits were recorded on 15 randomly selected individuals from the Greek population twice by the first author (RE) and twice by Weronika Flis, from Jagiellonian University, Poland. Intra- and inter-observer error was evaluated through Kendall’s W concordance coefficient, which ranges from 0 (no agreement) to 1 (perfect agreement) [16]. The intra- and inter-observer error analysis was performed using R Statistical Software [17] via the “irr” package [18].

## Results

Regarding the intra-observer error, the average Kendall’s W concordance coefficient was similar between the two observers. More specifically, for the first observer (RE), the average was 0.717, and for the second observer (WF), it was equal to 0.748. The traits with the lowest agreement (< 0.5) included “L4 Inferior Surface” (L4IS), “Humerus capitulum and trochlea” (HM04), “Ulna olecranon” (UL02), and “Radius head” (RD01). In contrast, those with the highest agreement (> 0.9) were 15, with some notable examples being the “Lamboid-pars asterica” (CRS06), “C3 Inferior Surface” (C3IS), “Ulna prox. articular facet” (UL01), “Femur lesser trochanter” (FM04), “Tibia condyles” (TB01), “1st rib’s Costal face” (RB101), and “Patella base” (PT02). With reference to the inter-observer error, the average Kendall’s W concordance coefficient was 0.615. The traits with the lowest agreement (< 0.5) included “Palatine-Posterior, Median” CRS01, “L2 body inferior surface and margin” L2IS, “Radius head” RD01, “Os coxa iliac tuberosity” OC01, “Os coxa ischial tuberosity” OC02, “Calcaneus tuberosity” CLN01, “Symphyseal topography” PSY02, “Symphyseal texture” PSY03, and “Sacroiliac texture” SAS01. The highest agreement (> 0.8) was achieved for eight traits, the “Coronal-Sagittal-pars bregmatica” (CRS03), “Coronal-pars pterica” (CRS04), “Lambdoid-pars asterica” (CRS06), “C6 body superior surface and margin” (C6SS), “S1 Superior Surface” (S1SS), “S1-S2 fusion” (S1S2F), “Proximal femur (trochanteric fossa)” (FM02), and “Patella (base)” (PT02). The average total Kendall’s W, calculated by all four observations, was 0.498. The results for intra- and inter-observed error are given in detail in Table S2 in Supplementary Material.

The results of the DRNNAGE software validity when utilizing the ensembled randomized network (ERN) option for age-at-death estimation are presented in Table 2. When combining all anatomical regions, females were more frequently correctly aged than males (52% vs. 41.3%). Regarding the anatomical regions individually, for the pooled sample, the cranial sutures exhibited the highest validity (82.9%), followed by the clavicle and 1st rib (79.9%), the acetabulum (79.3%), and the pubic symphysis (79.2%). In contrast, the vertebrae showed the lowest validity (42.6%). The above pattern also characterized males and females when each sex was examined separately, as well as the two broad age groups (below and over 50 years) when studied independently. It is striking that individuals over 50 years old were classified in their correct age group with much higher frequency (64.4–94.9%) compared to individuals under 50 years old (6.2–67.9%) in the majority of anatomical regions. Regarding the vertebrae and lower limb, although individuals over 60 years old expressed higher validity compared to those younger than 49 years old, the age group 50–59 years old showed extremely low validity. Furthermore, the upper limb appeared to under-perform as an age predictor in the ages between 40 and 59 years old, while expressing higher validity in the other age groups. Finally, the cranial sutures performed much better in the younger age groups, presenting a downward trend with increasing age.

**Table 2:** DRNNAGE validity when utilizing the ensembled randomized network (ERN) for age-at-death estimation

	Anatomical regions								
	All regions	Cranial sutures	Vertebrae	Upper limb	Lower limb	Clavicle and 1st rib	Pubic symphysis	Sacroiliac joint	Acetabulum
Pooled	0.461	0.829	0.426	0.699	0.539	0.799	0.792	0.654	0.793
Female	0.520	0.827	0.485	0.704	0.551	0.832	0.770	0.701	0.804
Male	0.413	0.832	0.378	0.694	0.529	0.773	0.807	0.615	0.783
Individuals < 50 yo	0.110	0.988	0.062	0.537	0.134	0.679	0.515	0.207	0.573
Individuals > 50 yo	0.672	0.737	0.644	0.796	0.781	0.868	0.949	0.932	0.912
Female < 50 yo	0.176	1.000	0.147	0.500	0.118	0.781	0.462	0.235	0.576
Female > 50 yo	0.703	0.734	0.667	0.813	0.781	0.857	0.938	0.952	0.922
Male < 50 yo	0.063	0.978	0.000	0.563	0.146	0.609	0.550	0.188	0.596
Male > 50 yo	0.644	0.740	0.625	0.781	0.781	0.877	0.957	0.913	0.904
Individuals 19–29 yo	0.000	0.957	0.000	0.739	0.087	0.773	0.294	0.130	0.348
Individuals 30–39 yo	0.143	1.000	0.000	0.619	0.238	0.600	0.500	0.095	0.500
Individuals 40–49 yo	0.182	1.000	0.152	0.394	0.121	0.677	0.645	0.273	0.781
Individuals 50–59 yo	0.063	1.000	0.063	0.344	0.063	0.781	0.857	0.719	0.906
Individuals 60–69 yo	0.487	0.846	0.405	0.718	0.897	0.974	1.000	1.000	0.949
Individuals 70–79 yo	1.000	0.676	1.000	1.000	1.000	0.879	0.963	1.000	1.000
Individuals 80–89 yo	1.000	0.581	0.968	1.000	1.000	0.839	0.926	0.929	0.903
Individuals > 90 yo	1.000	0.000	1.000	1.000	0.833	0.500	0.800	1.000	0.167

Regarding the separate anatomical regions, the greatest bias was found in the vertebrae, lower limb, pubic symphysis, and sacroiliac joint (Table 3). The lowest bias was seen in the clavicle and first rib while for the remaining anatomical regions, bias depended largely on the demographics under study. When using the vertebrae, upper limbs, lower limbs, pubic symphysis, and sacroiliac joint, DRNNAGE always overaged individuals, irrespective of their sex and age group. This is interesting because most existing methods tend to overage younger adults and underage older ones. As noted, except for the individuals under 50 years old, the method under-predicted the age-at-death of females more than males when analyzing the cranial sutures. The same applies to individuals over 50 years old when analyzing the acetabulum. Similarly, the method under-predicted the age-at-death of females over 50 years old regarding the clavicle/1st rib. In all other cases, an over-prediction of an individual’s chronological age was observed. The vertebrae exhibited the highest over-prediction of an individual’s chronological age, while the clavicle/1st rib and the acetabulum showed the lowest, followed by the pubic symphysis. Furthermore, except for the pubic symphysis, where the opposite applied, the bias was higher for males and individuals under 50 years old compared to females and individuals over 50 years old. However, for the acetabulum, the differences between the sexes were small. In addition, regarding the overaging of younger adults and the underaging of older ones, for the acetabulum there was a strong overaging of individuals younger than 50 years and a slight underaging of those older than 50 years, in the clavicle and 1st rib this pattern was only seen among females, while in cranial sutures the overaging of young adults was slight but the underaging of older ones very pronounced. Finally, when using a decade-based segmentation, the results showed an underestimation of age-at-death for individuals over 70 years old in most anatomical regions. It is worth mentioning that the cranial sutures expressed an upward trend in underestimation of age-at-death for individuals over 40 years old.

**Table 3:** Bias (measured in years) of the DRNNAGE software utilizing the ensembled randomized network (ERN) to predict age-at-death

	Anatomical regions								
	All regions	Cranial sutures	Vertebrae	Upper limb	Lower limb	Clavicle and 1st rib	Pubic symphysis	Sacroiliac joint	Acetabulum
Pooled	13.373	−10.833	17.860	11.313	15.534	3.458	9.675	13.554	4.784
Female	12.384	−12.829	16.286	10.555	14.885	2.226	11.803	11.833	4.417
Male	14.174	−9.189	19.143	11.926	16.060	4.441	8.230	14.980	5.080
Individuals < 50 yo	19.458	3.088	28.517	15.863	27.047	9.483	21.013	27.432	15.546
Individuals > 50 yo	9.730	−18.962	11.466	8.589	8.643	0.002	3.278	4.932	−1.727
Female < 50 yo	17.985	0.752	25.363	15.616	26.871	8.304	22.918	25.668	16.461
Female > 50 yo	9.408	−20.044	11.387	7.867	8.518	−0.861	5.782	4.366	−1.793
Male < 50 yo	20.502	4.815	30.798	16.038	27.172	10.302	19.776	28.681	15.564
Male > 50 yo	10.013	−18.013	11.536	9.222	8.753	0.747	1.537	5.449	−1.670
Individuals 19–29 yo	17.868	10.244	31.363	11.035	28.223	7.574	30.693	31.863	18.914
Individuals 30–39 yo	17.584	5.143	28.013	13.239	25.577	5.739	25.058	30.507	16.445
Individuals 40–49 yo	21.024	−1.134	26.606	19.448	27.062	12.923	18.666	22.837	13.853
Individuals 50–59 yo	21.415	−5.635	25.293	20.521	23.679	10.181	16.860	20.310	10.477
Individuals 60–69 yo	15.437	−12.709	18.144	14.639	14.667	5.901	9.145	10.831	4.357
Individuals 70–79 yo	6.892	−22.772	7.277	6.016	4.912	−2.129	0.546	0.347	−6.460
Individuals 80–89 yo	0.123	−28.910	0.929	−1.599	−2.746	−9.296	−8.453	−6.495	−10.289
Individuals > 90 yo	−11.804	−50.023	−10.706	−13.122	−14.816	−23.260	−19.371	−17.879	−20.941

The results of inaccuracy (Table 4) broadly agree with those for bias. The vertebrae, lower limbs, pubic symphysis, and sacroiliac joint, but also now the cranial sutures showed the highest inaccuracy rates. In contrast, the clavicle and first rib, followed by the acetabulum and upper limb had the lowest inaccuracy scores. The levels of inaccuracy were largely comparable between males and females. However, inaccuracy was generally higher for individuals younger than 50 years old compared to those older than 50, in agreement with the results of Table 2. The only exception to this pattern was cranial sutures, where the opposite was observed, but also the clavicle and first rib where the difference between age groups was very small.

**Table 4:** Inaccuracy (measured in years) of the DRNNAGE software utilizing the ensembled randomized network (ERN) to predict age-at-death

	Anatomical regions								
	All regions	Cranial sutures	Vertebrae	Upper limb	Lower limb	Clavicle and 1st rib	Pubic symphysis	Sacroiliac joint	Acetabulum
Pooled	14.524	16.700	19.073	13.247	17.255	10.719	14.401	16.839	11.784
Female	13.342	17.609	17.059	12.701	16.456	10.178	15.455	15.072	11.519
Male	15.482	15.951	20.715	13.688	17.903	11.151	13.685	18.304	11.999
Individuals < 50 yo	19.726	9.956	28.517	17.029	27.196	11.543	21.013	27.566	15.988
Individuals > 50 yo	11.411	20.638	13.407	10.875	11.305	10.247	10.671	10.175	9.096
Female < 50 yo	17.985	10.628	25.363	17.564	26.871	9.401	22.918	25.992	16.793
Female > 50 yo	10.875	21.318	12.577	10.118	10.922	10.572	11.413	9.180	8.800
Male < 50 yo	20.960	9.459	30.798	16.651	27.425	13.032	19.776	28.681	16.103
Male > 50 yo	11.881	20.042	14.133	11.740	11.641	9.965	10.154	11.085	9.357
Individuals 19–29 yo	17.868	10.244	31.363	11.035	28.223	8.369	30.693	31.863	18.914
Individuals 30–39 yo	18.630	7.951	28.013	15.181	26.156	10.549	25.058	30.507	17.313
Individuals 40–49 yo	21.024	10.366	26.606	21.110	27.062	14.195	18.666	23.171	14.445
Individuals 50–59 yo	21.415	10.780	25.293	20.521	23.679	11.592	17.769	20.310	10.816
Individuals 60–69 yo	15.437	14.862	18.144	15.055	14.667	10.958	11.562	11.296	5.921
Individuals 70–79 yo	7.005	22.772	8.953	6.215	5.181	7.051	4.512	3.189	7.846
Individuals 80–89 yo	2.857	28.910	3.398	3.158	2.989	9.296	8.453	6.495	10.782
Individuals > 90 yo	11.804	50.023	10.706	13.122	14.816	23.260	19.371	17.879	20.941

As mentioned above, the DRNNAGE software has four available network algorithms to create regression models for age-at-death estimation: randomized network algorithm, ensembled randomized network, and two different ensemble autoencoder networks. The comparison of the available network algorithms showed no major differences in the DRNNAGE validity; instead, all networks gave the same broad patterns (Tables S3-S11). However, the bias and inaccuracy when utilizing the ensembled autoencoder (S) network were higher in most anatomical regions and sample groups, a pattern particularly visible for the cranial sutures. In addition, the bias for cranial sutures when using this network was always negative, irrespective of the age group under study.

## Discussion

Any forensic anthropological method must be tested for repeatability, reproducibility, and accuracy before its use is generalized and it becomes admissible to legal contexts [19, 20]. According to the DRNNAGE software developers [14], all skeletal traits employed in their method presented a very high (average value of 0.907) and statistically significant concordance coefficient regarding intra-observer error, with only exception the “Radius head” (RD01) and “Femur head” (FM01). In contrast, the average intra-observer error concordance coefficient in our study was 0.717 for the first observer and 0.748 for the second. There was great variability in the coefficient’s values among different traits; thus, certain traits should be favored and others should be used cautiously or be avoided altogether.

Inter-observer error, as expected, showed an even smaller concordance coefficient (average value 0.615). Once again, some traits showed much higher reproducibility than others and should be thus preferred, while others should be avoid. At this point, we must stress that DRNNAGE involved many traits that are binary-coded. Therefore, we would have expected better reproducibility and repeatability results since methods using a narrower scale of categories produce greater agreement among researchers [20].

The use of different network algorithms to predict age-at-death had a minimal impact on the results, though in our sample, the ensemble autoencoder S network showed higher bias and inaccuracy values, so any of the remaining three options should be preferred.

The validity of DRNNAGE was overall average in the modern Greek assemblage, both for males and females. Very interestingly, this software showed a high validity for individuals older than 50 years old, which is often a problematic category when using “traditional” skeletal age-at-death estimation methods, whereas the results were very poor for those younger than 50. Surprisingly, the cranial sutures exhibited the highest validity, even for younger adults. Among the remaining anatomical areas, those with high validity included the clavicle and 1st rib, the acetabulum, and the pubic symphysis, while the lowest validity was achieved by the vertebrae. Similarly, the greatest bias and inaccuracy were found in the vertebrae, and the lowest in the clavicle and first rib. The above observations are corroborated by the analyses performed on the decade based segmented sample. However, it is important to note that the sample size for the group of individuals older than 90 years old is very small and the respective results should be interpreted with caution.

These results are partly in agreement with another validation study performed on the Athens Collection, employing traditional age-at-death estimation methods focused on the public symphysis, iliac auricular surface, and cranial sutures [13]. In specific, Xanthopoulou and colleagues found that the iliac auricular surface when recorded using the Lovejoy et al. [21] method works satisfactorily for all age groups; cranial sutures and the pubic symphysis were found to perform satisfactorily for individuals younger than 50 years old but poorly for older ones, while the iliac auricular surface recorded using the Buckberry and Chamberlain [22] method gave the most accurate results for individuals older than 50 years. The differences in the performance of the DRNNAGE software compared to these traditional methods, even when focusing on the same anatomical areas, must be attributed to the different ways in which skeletal changes are recorded in each method but also to their different statistical treatment for age prediction.

The vertebrae, and more specifically the fusion of the superior and inferior epiphyses, have been established and popularized over the years as a viable method of skeletal age estimation in teenagers and young adults [23–25]. As a rather recent example, Albert et al. [26] achieved over 78% classification accuracy when studying 57 individuals aged 14–27 years. For older adults, several studies have shown that osteophyte formation could be useful for estimating the age-at-death [27–29]. Very recently, Sluis and colleagues [30] tested three methods based on osteophyte formation on 88 individuals from the Middenbeemster cemetery and achieved over 72.73% classification accuracy. The DRNNAGE scoring system for vertebrae covers the whole spectrum from the fusion of the epiphyseal ring to the formation of lipping. However, all these major changes from ring fusion to lipping are covered within merely three stages, which do not express sufficiently different degrees of lipping that are anticipated in middle aged and older adults Therefore, the low validity and high bias and inaccuracy observed in our study may be due to the skewed age-at-death distribution towards older people in the Athens Collection.

The cranial suture closure pattern has been studied as a potential age-at-death predictor for nearly a century [31]. Several studies since then have proposed variants of different recording schemes for age prediction based on different sutures and suture combinations [32–34]. In parallel, numerous validation studies have stressed the poor performance of this method (e.g. [35]). The high validity in age estimation achieved in the Athens Collection for individuals younger than 69 years old via DRNNAGE is thus surprising; however, it also aligns with a recent review that stressed the potential of this anatomical area as a useful indicator for age estimation [36].

With regard to the other anatomical areas that showed high accuracies, Kunos et al. [37] were the first to use the first rib for age-at-death estimation because it is easily identifiable, not influenced by mechanical stress in the same manner as the lower ribs and exhibits a prolonged span of remodeling into the eighth decade. In a recent study on 260 skeletons from the Raymond A. Dart Collection of Human Skeletons, Jooste and Steyn [38] concluded that the first rib can be used to make age-at-death predictions but should ideally be used in combination with other skeletal traits. The DRNNAGE software combines the first rib with the clavicle, which has the potential to aid age estimates beyond the traditional “mature adult” age category (> 46 years) [39], while its usefulness in providing precise age estimations between the ages of 16 and 30 years has been identified by several studies [2, 40–43]. Therefore, our results from the Athens Collection, showing high age-group classification rates for the clavicle and first rib are not surprising.

The acetabulum and pubic symphysis were the remaining two anatomical areas that gave overall high validity values in the Athens Collection. The adult human pelvis has been among the most useful areas for age-at-death estimation and contains different anatomical structures that have been used for this purpose: pubic symphysis, auricular surface, and acetabulum. Bony degenerative changes in these regions have been shown to correlate with age [44–46]. Although several studies have demonstrated that relevant methods can most commonly support age estimates between the late teens and 50–60 years, where the observations of the progressive degenerative changes reach their peak breakdown and plateau [21, 22, 47, 48], in the present study the DRNNAGE software performs better for individuals over 49 years old. In what concerns the epiphyseal union at the upper and lower limbs, this has been established as a viable method of skeletal age estimation in teenagers and young adults [42]. For more mature adults, the most commonly used age estimation methods based on the upper and lower limbs focus on degenerative changes on the articular surfaces [21, 47–49]. This latter approach is also the one followed at DRNNAGE. The upper and lower limbs showed a generally moderate validity in skeletal age estimation for individuals aged from 19 to 59 years old at the Athens Collection, with the exception of the upper limb performance for the age group 19–29 years old. This may be linked to the fact that articular changes (usually osteophytes and porosity) are strongly associated with mechanical stress linked to daily occupations but also body weight and other factors, besides age [50].

Finally, for a more direct comparison with the performance of the DRNNAGE software as reported by its developers [14], the variable combinations described in their work were also tested. The results of these analyses are provided in Supplementary Material (Table S12 and Figure S1). According to the comparison, the anatomical regions of the sutures, the 1st rib, and the pubic symphysis showed similar validity values in both studies, while major differences in validity were observed for the variable combinations regarding axial, appendicular, sacroiliac, and standard traits. Specifically, the DRNNAGE models severely underperformed in the Athens Collection. Similarly, the anatomical regions of the clavicle and the acetabulum underperformed in the Athens Collection sample; however, the observed differences in validity values were moderate.

Although it has been often supported in the literature that using a multivariate approach for skeletal age estimation is more proper than using any single method alone [51, 52], in the present study the lowest classification rate was obtained when combining all available anatomical regions. It is well known that the aging process is controlled by various internal and external factors [6, 7], which affect different anatomical areas differently and this can become a source of bias in age estimation. Furthermore, the validity of skeletal age estimation can be affected by within and between individuals and populations variation in the rate of senescence [53]. The DRNNAGE software was trained utilizing skeletal collections hosted at the University of Coimbra (CISC, XXI-ISC) which are composed of individuals of Portuguese ancestry. Furthermore, the age-at-death distribution of the reference sample used by the DRNNAGE developers was homogeneous across the represented age-at-death span, whereas the proportion of older individuals is higher in the Athens Collection. Therefore, the validity loss observed could be attributed to either population specificity or the different age-at-death distributions of the utilized samples.

In conclusion, the DRNNAGE software produced partly accurate age-at-death predictions in a modern Greek assemblage. This method was particularly successful for males and females older than 50 years, but it performed poorly for those younger than this threshold, with only exception the use of cranial suture closure. Moreover, different anatomical areas showed very different repeatability, reproducibility, and validity. Further evaluation studies in different assemblages are necessary in order to test the performance of this software more broadly.

### Supplementary information

ESM 1 (DOC 370 kb)

## Data Availability

The datasets generated during and/or analyzed during the current study are available from the corresponding author on reasonable request.

## Footnotes

1 At the time of writing this paper, the default parameter values for all models are as follows: layer size, 32; network depth, 8; Gaussian noise, 1; uncertainty level, 0.1; variance model exponent, 1; and RNG seed, 99,676.
