# Supplementary material for: Testing the accuracy of the DRNNAGE software for age estimation in a modern Greek sample
Source: Int J Legal Med. 2023 Nov 25;138(3):917–26. doi: 10.1007/s00414-023-03129-4 (PMC11003917; doi:10.1007/s00414-023-03129-4)

**Table S1. Skeletal traits and regions as described in the DRNNAGE software**

| Region | Skeletal traits | | |
| --- | --- | --- | --- |
|  | Abbreviation | Description | |
| Sutures | CRS01 | | Palatine (posterior median) |
|  | CRS02L | | "Palatine (transverse left)" |
|  | CRS02R | | "Palatine (transverse right)" |
|  | CRS03 | | Coronal - Sagittal (pars bregmatica) |
|  | CRS04L | | "Coronal (pars pterica left)" |
|  | CRS04R | | "Coronal (pars pterica right)" |
|  | CRS05 | | Sagittal - Lambdoid (pars lambdica) |
|  | CRS06L | | "Lambdoid (pars asterica left)" |
|  | CRS06R | | "Lambdoid (pars asterica right)" |
| Vertebrae | C3IS | | C3 body inferior surface and margin |
|  | C4SS | | C4 body superior surface and margin |
|  | C4IS | | C4 body inferior surface and margin |
|  | C5SS | | C5 body superior surface and margin |
|  | C5IS | | C5 body inferior surface and margin |
|  | C6SS | | C6 body superior surface and margin |
|  | C6IS | | C6 body inferior surface and margin |
|  | C7SS | | C7 body superior surface and margin |
|  | L1IS | | L1 body inferior surface and margin |
|  | L2SS | | L2 body superior surface and margin |
|  | L2IS | | L2 body inferior surface and margin |
|  | L3SS | | L3 body superior surface and margin |
|  | L3IS | | L3 body inferior surface and margin |
|  | L4SS | | L4 body superior surface and margin |
|  | L4IS | | L4 body inferior surface and margin |
|  | L5SS | | L5 body superior surface and margin |
|  | S1SS | | S1 body superior surface and margin |
|  | S1S2F | | S1-S2 fusion |
| Limbs | SC01 | | Scapula (glenoid fossa) Joint Shoulder |
|  | HM01 | | Proximal humerus (head) Joint Shoulder |
|  | HM02 | | Proximal humerus (lesser tubercle) Musculoskeletal Shoulder |
|  | HM03 | | Proximal humerus (greater tubercle) Musculoskeletal Shoulder |
|  | HM04 | | Distal humerus (trochlea and capitulum) Joint Elbow |
|  | HM05 | | Distal humerus (medial epicondyle) Musculoskeletal Elbow |
|  | HM06 | | Distal humerus (lateral epicondyle) Musculoskeletal Elbow |
|  | UL01 | | Proximal ulna (articular facets) Joint Elbow |
|  | UL02 | | Proximal ulna (olecranon) Musculoskeletal Elbow |
|  | RD01 | | Proximal radius (head) Joint Elbow |
|  | RD02 | | Proximal radius (radial tuberosity) Musculoskeletal Elbow |
|  | OC01 | | Os coxa (iliac tuberosity) Musculoskeletal Hip |
|  | OC02 | | Os coxa (ischial tuberosity) Musculoskeletal Hip |
|  | OC03 | | Os coxa (acetabulum) Joint Hip |
|  | FM01 | | Proximal femur (head) Joint Hip |
|  | FM02 | | Proximal femur (trochanteric fossa) Musculoskeletal Hip |
|  | FM03 | | Proximal femur (greater trochanter) Musculoskeletal Hip |
|  | FM04 | | Proximal femur (lesser trochanter) Musculoskeletal Hip |
|  | FM05 | | Distal femur (condyles) Joint Knee |
|  | TB01 | | Proximal tibia (condyles) Joint Knee |
|  | PT01 | | Patella (articular facets) Joint Knee |
|  | PT02 | | Patella (base) Musculoskeletal Knee |
|  | CLN01 | | Patella (calcaneal tuberosity, superior) Musculoskeletal Ankle |
| Clavicle and 1^st^ rib | CLV01 | | Sternal end |
|  | CLV02 | | Acromial end |
|  | RB101 | | Costal face |
|  | RB102 | | Tubercule |
| Os oxae | PSY01 | | Rim |
|  | PSY02 | | Topography |
|  | PSY03 | | Texture |
|  | SAS01 | | Texture |
|  | SAS02 | | Margin |
|  | IAS01 | | Texture |
|  | IAS02 | | Margin |
|  | ACT01 | | Rim |
|  | ACT02 | | Posterior horn |
|  | ACT03 | | Fossa |

**Table S2.** Intra- and Inter- observer error: Kendall's W results.

| **Variables** | **First observer**  **(intra-observer)** | | **Second observer**  **(inter-, intra-observer)** | | | | **Total** | |
| --- | --- | --- | --- | --- | --- | --- | --- | --- |
|  | **N** | **Kendall's W** | **N** | **Kendall's W** | **N** | **Kendall's W** | **N** |  |
| **CRS01** | 15 | 0.825 | 15 | 0.450 | 15 | 0.814 | 15 |  |
| **CRS02L** | 15 | 0.825 | 15 | 0.560 | 15 | 0.800 | 15 |  |
| **CRS02R** | 15 | 0.825 | 15 | 0.560 | 15 | 0.800 | 15 |  |
| **CRS03** | 15 | 0.537 | 15 | 1.000 | 15 | 0.644 | 15 |  |
| **CRS04L** | 15 | 0.788 | 15 | 0.877 | 15 | 0.800 | 15 |  |
| **CRS04R** | 15 | 0.788 | 15 | 0.830 | 15 | 0.804 | 15 |  |
| **CRS05** | 15 | 0.800 | 15 | 0.714 | 15 | 0.867 | 15 |  |
| **CRS06L** | 15 | 0.938 | 15 | 0.882 | 15 | 0.936 | 15 |  |
| **CRS06R** | 15 | 0.882 | 15 | 0.882 | 15 | 0.882 | 15 |  |
| **C3IS** | 10 | 1.000 | 10 | 0.758 | 10 | 1.000 | 10 |  |
| **C4SS** | 10 | 0.800 | 10 | 0.694 | 10 | 0.761 | 10 |  |
| **C4IS** | 9 | 0.500 | 9 | 0.500 | 10 | 0.425 | 9 |  |
| **C5SS** | 10 | 0.500 | 10 | 0.500 | 10 | 0.668 | 10 |  |
| **C5IS** | 9 | 0.895 | 10 | 0.711 | 10 | 0.915 | 9 |  |
| **C6SS** | 9 | 0.529 | 9 | 0.875 | 10 | 0.561 | 9 |  |
| **C6IS** | 10 | 0.500 | 10 | 0.500 | 10 | 0.579 | 10 |  |
| **C7SS** | 9 | 0.500 | 6 | 0.700 | 6 | 0.605 | 6 |  |
| **L1IS** | 11 | 0.574 | 13 | 0.526 | 13 | 0.607 | 11 |  |
| **L2SS** | 9 | 0.643 | 12 | 0.603 | 12 | 0.656 | 9 |  |
| **L2IS** | 13 | 0.738 | 13 | 0.433 | 13 | 0.708 | 13 |  |
| **L3SS** | 11 | 0.684 | 11 | 0.653 | 11 | 0.733 | 10 |  |
| **L3IS** | 9 | 0.679 | 11 | 0.640 | 11 | 0.747 | 9 |  |
| **L4SS** | 13 | 0.705 | 13 | 0.699 | 13 | 0.650 | 13 |  |
| **L4IS** | 11 | 0.395 | 11 | 0.658 | 12 | 0.380 | 10 |  |
| **L5SS** | 12 | 0.737 | 12 | 0.581 | 12 | 0.748 | 12 |  |
| **S1SS** | 15 | 0.690 | 15 | 0.858 | 15 | 0.725 | 15 |  |
| **S1S2F** | 15 | 1 | 15 | 1 | 15 | 1 | 15 |  |
| **SC01** | 13 | 0.824 | 14 | 0.761 | 14 | 0.846 | 13 |  |
| **HM01** | 14 | 0.689 | 14 | 0.613 | 14 | 0.723 | 14 |  |
| **HM02** | 13 | 0.500 | 13 | 0.500 | 13 | 0.586 | 13 |  |
| **HM03** | 12 | 0.500 | 13 | 0.500 | 13 | 0.630 | 12 |  |
| **HM04** | 11 | 0.412 | 12 | 0.579 | 12 | 0.444 | 11 |  |
| **HM05** | 13 | 0.688 | 13 | 0.596 | 13 | 0.707 | 13 |  |
| **HM06** | 12 | 1 | 13 | 0.500 | 13 | 0.927 | 12 |  |
| **UL01** | 14 | 1 | 15 | 0.690 | 15 | 1.000 | 14 |  |
| **UL02** | 14 | 0.446 | 14 | 0.500 | 14 | 0.545 | 13 |  |
| **RD01** | 13 | 0.362 | 15 | 0.400 | 15 | 0.439 | 13 |  |
| **RD02** | 15 | 0.500 | 15 | 0.614 | 15 | 0.527 | 15 |  |
| **OC01** | 15 | 0.500 | 15 | 0.450 | 15 | 0.566 | 15 |  |
| **OC02** | 14 | 0.500 | 14 | 0.446 | 14 | 0.558 | 14 |  |
| **OC03** | 14 | 1 | 14 | 0.500 | 15 | 1.000 | 14 |  |
| **FM01** | 14 | 1 | 15 | 0.500 | 15 | 1.000 | 14 |  |
| **FM02** | 13 | 0.500 | 15 | 1 | 15 | 0.500 | 13 |  |
| **FM03** | 14 | 1 | 15 | 0.500 | 15 | 1.000 | 14 |  |
| **FM04** | 14 | 1.000 | 15 | 0.740 | 15 | 1.000 | 14 |  |
| **FM05** | 15 | 0.740 | 15 | 0.588 | 15 | 0.745 | 15 |  |
| **TB01** | 11 | 1.000 | 14 | 0.565 | 15 | 1.000 | 11 |  |
| **PT01** | 7 | 0.500 | 6 | 0.654 | 7 | 0.667 | 6 |  |
| **PT02** | 7 | 1.000 | 6 | 1.000 | 6 | 1.000 | 6 |  |
| **CLN01** | 13 | 0.712 | 14 | 0.395 | 15 | 0.800 | 13 |  |
| **CLV01** | 15 | 0.674 | 15 | 0.603 | 15 | 0.739 | 15 |  |
| **CLV02** | 15 | 0.500 | 15 | 0.656 | 15 | 0.644 | 15 |  |
| **RB101** | 12 | 0.907 | 11 | 0.643 | 11 | 0.904 | 11 |  |
| **RB102** | 12 | 1 | 12 | 0.500 | 12 | 1.000 | 12 |  |
| **PSY01** | 11 | 0.630 | 12 | 0.619 | 12 | 0.711 | 11 |  |
| **PSY02** | 12 | 0.750 | 12 | 0.457 | 12 | 0.784 | 12 |  |
| **PSY03** | 12 | 0.560 | 12 | 0.436 | 12 | 0.559 | 12 |  |
| **SAS01** | 15 | 0.674 | 15 | 0.326 | 15 | 0.708 | 15 |  |
| **SAS02** | 15 | 1 | 15 | 0.500 | 15 | 1.000 | 15 |  |
| **IAS01** | 15 | 0.500 | 15 | 0.656 | 15 | 0.700 | 15 |  |
| **IAS02** | 15 | 1 | 15 | 0.500 | 15 | 1.000 | 15 |  |
| **ACT01** | 15 | 0.736 | 15 | 0.599 | 15 | 0.787 | 15 |  |
| **ACT02** | 15 | 0.500 | 15 | 0.674 | 15 | 0.454 | 15 |  |
| **ACT03** | 14 | 0.789 | 14 | 0.718 | 14 | 0.870 | 14 |  |

**Table S3.** DRNNAGE validity when utilizing the randomized network (RN) for age-at-death estimation.

|  | **Anatomical regions** | | | | | | | |
| --- | --- | --- | --- | --- | --- | --- | --- | --- |
|  | **All Regions** | **Cranial Sutures** | **Vertebrae** | **Upper Limb** | **Lower Limb** | **Clavicle and 1^st^ Rib** | **Pubic Symphysis** | **Sacroiliac Joint** |
| **Pooled** | 0.452 | 0.829 | 0.449 | 0.667 | 0.525 | 0.804 | 0.803 | 0.668 |
| **Female** | 0.510 | 0.816 | 0.495 | 0.663 | 0.551 | 0.832 | 0.770 | 0.691 |
| **Male** | 0.405 | 0.840 | 0.412 | 0.669 | 0.504 | 0.782 | 0.826 | 0.650 |
| **Pooled <50 yrs** | 0.110 | 0.988 | 0.062 | 0.488 | 0.098 | 0.679 | 0.545 | 0.220 |
| **Pooled >50 yrs** | 0.657 | 0.737 | 0.681 | 0.774 | 0.781 | 0.875 | 0.949 | 0.947 |
| **Female <50 yrs** | 0.176 | 1.000 | 0.147 | 0.441 | 0.118 | 0.781 | 0.462 | 0.206 |
| **Female >50 yrs** | 0.688 | 0.719 | 0.683 | 0.781 | 0.781 | 0.857 | 0.938 | 0.952 |
| **Male <50 yrs** | 0.063 | 0.978 | 0.000 | 0.521 | 0.083 | 0.609 | 0.600 | 0.229 |
| **Male >50 yrs** | 0.630 | 0.753 | 0.681 | 0.767 | 0.781 | 0.890 | 0.957 | 0.942 |

**Table S4.** DRNNAGE validity when utilizing the ensembled Autoencoder (U) network for age-at-death estimation.

|  | **Anatomical regions** | | | | | | | |
| --- | --- | --- | --- | --- | --- | --- | --- | --- |
|  | **All Regions** | **Cranial Sutures** | **Vertebrae** | **Upper Limb** | **Lower Limb** | **Clavicle and 1^st^ Rib** | **Pubic Symphysis** | **Sacroiliac Joint** |
| **Pooled** | 0.461 | 0.820 | 0.468 | 0.680 | 0.539 | 0.776 | 0.803 | 0.692 |
| **Female** | 0.520 | 0.806 | 0.515 | 0.673 | 0.561 | 0.800 | 0.770 | 0.732 |
| **Male** | 0.413 | 0.832 | 0.429 | 0.686 | 0.521 | 0.756 | 0.826 | 0.658 |
| **Pooled <50 yrs** | 0.110 | 0.988 | 0.086 | 0.537 | 0.146 | 0.654 | 0.545 | 0.268 |
| **Pooled >50 yrs** | 0.672 | 0.723 | 0.696 | 0.766 | 0.774 | 0.846 | 0.949 | 0.955 |
| **Female <50 yrs** | 0.176 | 1.000 | 0.147 | 0.500 | 0.147 | 0.750 | 0.462 | 0.324 |
| **Female >50 yrs** | 0.703 | 0.703 | 0.714 | 0.766 | 0.781 | 0.825 | 0.938 | 0.952 |
| **Male <50 yrs** | 0.063 | 0.978 | 0.043 | 0.563 | 0.146 | 0.587 | 0.600 | 0.229 |
| **Male >50 yrs** | 0.644 | 0.740 | 0.681 | 0.767 | 0.767 | 0.863 | 0.957 | 0.957 |

**Table S5.** DRNNAGE validity when utilizing the ensembled Autoencoder (S) network for age-at-death estimation.

|  | **Anatomical regions** | | | | | | | |
| --- | --- | --- | --- | --- | --- | --- | --- | --- |
|  | **All Regions** | **Cranial Sutures** | **Vertebrae** | **Upper Limb** | **Lower Limb** | **Clavicle and 1^st^ Rib** | **Pubic Symphysis** | **Sacroiliac Joint** |
| **Pooled** | 0.429 | 0.788 | 0.412 | 0.667 | 0.493 | 0.804 | 0.754 | 0.636 |
| **Female** | 0.480 | 0.735 | 0.485 | 0.684 | 0.500 | 0.842 | 0.689 | 0.680 |
| **Male** | 0.388 | 0.832 | 0.353 | 0.653 | 0.488 | 0.773 | 0.798 | 0.598 |
| **Pooled <50 yrs** | 0.110 | 0.988 | 0.086 | 0.561 | 0.098 | 0.718 | 0.485 | 0.232 |
| **Pooled >50 yrs** | 0.620 | 0.672 | 0.607 | 0.730 | 0.730 | 0.853 | 0.906 | 0.886 |
| **Female <50 yrs** | 0.176 | 0.971 | 0.176 | 0.529 | 0.088 | 0.844 | 0.423 | 0.265 |
| **Female >50 yrs** | 0.641 | 0.609 | 0.651 | 0.766 | 0.719 | 0.841 | 0.833 | 0.905 |
| **Male <50 yrs** | 0.063 | 1.000 | 0.021 | 0.583 | 0.104 | 0.630 | 0.525 | 0.208 |
| **Male >50 yrs** | 0.603 | 0.726 | 0.569 | 0.699 | 0.740 | 0.863 | 0.957 | 0.870 |

**Table S6*.*** Bias measured in years of the DRNNAGE software utilizing the randomized network (RN) to predict age-at-death.

|  | **Anatomical regions** | | | | | | |
| --- | --- | --- | --- | --- | --- | --- | --- |
|  | **Cranial Sutures** | **Vertebrae** | **Upper Limb** | **Lower Limb** | **Clavicle and 1^st^ Rib** | **Pubic Symphysis** | **Sacroiliac Joint** |
| **Pooled** | -10.010 | 17.964 | 12.002 | 15.811 | 3.415 | 9.632 | 13.589 |
| **Female** | -12.032 | 16.067 | 11.325 | 15.129 | 2.104 | 11.722 | 12.200 |
| **Male** | -8.345 | 19.511 | 12.550 | 16.363 | 4.462 | 8.213 | 14.740 |
| **Pooled <50 yrs** | 4.127 | 28.492 | 16.871 | 27.641 | 9.198 | 20.954 | 27.066 |
| **Pooled >50 yrs** | -18.266 | 11.648 | 9.087 | 8.730 | 0.099 | 3.245 | 5.216 |
| **Female <50 yrs** | 1.873 | 25.079 | 16.742 | 27.378 | 7.650 | 23.018 | 26.238 |
| **Female >50 yrs** | -19.419 | 11.203 | 8.446 | 8.622 | -0.713 | 5.603 | 4.624 |
| **Male <50 yrs** | 5.793 | 30.961 | 16.962 | 27.828 | 10.274 | 19.612 | 27.653 |
| **Male >50 yrs** | -17.255 | 12.037 | 9.649 | 8.825 | 0.800 | 1.604 | 5.757 |

**Table S7.** Bias measured in years of the DRNNAGE software utilizing the ensembled Autoencoder (U) network to predict age-at-death.

|  | **Anatomical regions** | | | | | | |
| --- | --- | --- | --- | --- | --- | --- | --- |
|  | **Cranial Sutures** | **Vertebrae** | **Upper Limb** | **Lower Limb** | **Clavicle and 1^st^ Rib** | **Pubic Symphysis** | **Sacroiliac Joint** |
| **Pooled** | -11.593 | 17.687 | 11.208 | 15.258 | 3.801 | 9.500 | 13.098 |
| **Female** | -13.413 | 15.796 | 10.489 | 14.532 | 2.782 | 11.698 | 11.477 |
| **Male** | -10.095 | 19.229 | 11.791 | 15.846 | 4.614 | 8.008 | 14.442 |
| **Pooled <50 yrs** | 2.809 | 27.937 | 15.605 | 26.786 | 10.064 | 20.714 | 26.551 |
| **Pooled >50 yrs** | -20.003 | 11.538 | 8.577 | 8.358 | 0.209 | 3.175 | 4.741 |
| **Female <50 yrs** | 0.780 | 24.360 | 15.318 | 26.494 | 9.422 | 22.782 | 25.111 |
| **Female >50 yrs** | -20.954 | 11.175 | 7.924 | 8.177 | -0.591 | 5.694 | 4.119 |
| **Male <50 yrs** | 4.308 | 30.524 | 15.808 | 26.993 | 10.511 | 19.370 | 27.571 |
| **Male >50 yrs** | -19.170 | 11.855 | 9.149 | 8.516 | 0.899 | 1.422 | 5.308 |

**Table S8.** Bias measured in years of the DRNNAGE software utilizing the ensembled Autoencoder (S) network to predict age-at-death.

|  | **Anatomical regions** | | | | | | |
| --- | --- | --- | --- | --- | --- | --- | --- |
|  | **Cranial Sutures** | **Vertebrae** | **Upper Limb** | **Lower Limb** | **Clavicle and 1^st^ Rib** | **Pubic Symphysis** | **Sacroiliac Joint** |
| **Pooled** | -17.118 | 19.844 | 12.308 | 17.550 | 3.655 | 11.618 | 15.526 |
| **Female** | -19.023 | 17.859 | 11.558 | 17.014 | 2.711 | 14.514 | 13.840 |
| **Male** | -15.550 | 21.462 | 12.915 | 17.985 | 4.409 | 9.652 | 16.923 |
| **Pooled <50 yrs** | -7.399 | 29.143 | 14.827 | 28.193 | 8.703 | 21.351 | 28.003 |
| **Pooled >50 yrs** | -22.794 | 14.265 | 10.800 | 11.180 | 0.760 | 6.128 | 7.775 |
| **Female <50 yrs** | -10.033 | 25.420 | 14.680 | 28.144 | 8.519 | 23.408 | 26.291 |
| **Female >50 yrs** | -23.799 | 13.779 | 9.899 | 11.102 | -0.240 | 9.696 | 7.121 |
| **Male <50 yrs** | -5.451 | 31.836 | 14.931 | 28.229 | 8.831 | 20.014 | 29.216 |
| **Male >50 yrs** | -21.913 | 14.690 | 11.590 | 11.249 | 1.622 | 3.646 | 8.372 |

**Table S9.** Inaccuracy measured in years of the DRNNAGE software utilizing the randomized network (RN) to predict age-at-death.

|  | **Anatomical regions** | | | | | | |
| --- | --- | --- | --- | --- | --- | --- | --- |
|  | **Cranial Sutures** | **Vertebrae** | **Upper Limb** | **Lower Limb** | **Clavicle and 1^st^ Rib** | **Pubic Symphysis** | **Sacroiliac Joint** |
| **Pooled** | 16.376 | 18.947 | 13.792 | 17.533 | 10.854 | 14.307 | 16.733 |
| **Female** | 17.287 | 16.864 | 13.363 | 16.695 | 10.173 | 15.413 | 15.310 |
| **Male** | 15.625 | 20.646 | 14.140 | 18.211 | 11.398 | 13.556 | 17.912 |
| **Pooled <50 yrs** | 10.269 | 28.492 | 17.978 | 27.801 | 11.479 | 20.954 | 27.120 |
| **Pooled >50 yrs** | 19.941 | 13.221 | 11.287 | 11.387 | 10.496 | 10.558 | 10.280 |
| **Female <50 yrs** | 10.919 | 25.079 | 18.667 | 27.378 | 9.081 | 23.018 | 26.367 |
| **Female >50 yrs** | 20.669 | 12.430 | 10.545 | 11.020 | 10.728 | 11.294 | 9.343 |
| **Male <50 yrs** | 9.789 | 30.961 | 17.489 | 28.100 | 13.147 | 19.612 | 27.653 |
| **Male >50 yrs** | 19.303 | 13.912 | 11.938 | 11.709 | 10.295 | 10.045 | 11.136 |

**Table S10.** Inaccuracy measured in years of the DRNNAGE software utilizing the ensembled Autoencoder (U) network to predict age-at-death.

|  | **Anatomical regions** | | | | | | |
| --- | --- | --- | --- | --- | --- | --- | --- |
|  | **Cranial Sutures** | **Vertebrae** | **Upper Limb** | **Lower Limb** | **Clavicle and 1^st^ Rib** | **Pubic Symphysis** | **Sacroiliac Joint** |
| **Pooled** | 17.136 | 18.666 | 13.205 | 17.051 | 10.954 | 14.243 | 16.519 |
| **Female** | 18.123 | 16.570 | 12.728 | 16.198 | 10.357 | 15.509 | 14.895 |
| **Male** | 16.323 | 20.374 | 13.591 | 17.742 | 11.431 | 13.384 | 17.866 |
| **Pooled <50 yrs** | 9.678 | 27.937 | 16.741 | 26.939 | 12.020 | 20.714 | 26.687 |
| **Pooled >50 yrs** | 21.491 | 13.103 | 11.088 | 11.132 | 10.343 | 10.593 | 10.203 |
| **Female <50 yrs** | 10.600 | 24.360 | 17.198 | 26.494 | 10.164 | 22.782 | 25.393 |
| **Female >50 yrs** | 22.120 | 12.366 | 10.354 | 10.728 | 10.456 | 11.569 | 9.229 |
| **Male <50 yrs** | 8.996 | 30.524 | 16.418 | 27.255 | 13.311 | 19.370 | 27.603 |
| **Male >50 yrs** | 20.940 | 13.748 | 11.732 | 11.486 | 10.245 | 9.915 | 11.093 |

**Table S11.** Inaccuracy measured in years of the DRNNAGE software utilizing the ensembled Autoencoder (S) network to predict age-at-death.

|  | **Anatomical regions** | | | | | | |
| --- | --- | --- | --- | --- | --- | --- | --- |
|  | **Cranial Sutures** | **Vertebrae** | **Upper Limb** | **Lower Limb** | **Clavicle and 1^st^ Rib** | **Pubic Symphysis** | **Sacroiliac Joint** |
| **Pooled** | 21.841 | 20.420 | 14.121 | 18.654 | 10.914 | 15.729 | 17.799 |
| **Female** | 23.472 | 18.294 | 13.830 | 17.910 | 10.542 | 17.542 | 16.104 |
| **Male** | 20.498 | 22.152 | 14.356 | 19.257 | 11.211 | 14.499 | 19.204 |
| **Pooled <50 yrs** | 15.503 | 29,143 | 17.003 | 28.431 | 11.669 | 21.468 | 28.335 |
| **Pooled >50 yrs** | 25.542 | 15.186 | 12.396 | 12.803 | 10.481 | 12.492 | 11.254 |
| **Female <50 yrs** | 18.322 | 25.420 | 17.934 | 28.144 | 9.806 | 23.562 | 27.065 |
| **Female >50 yrs** | 26.208 | 14.448 | 11.650 | 12.474 | 10.916 | 14.282 | 10.188 |
| **Male <50 yrs** | 13.419 | 31.836 | 16.343 | 28.634 | 12.965 | 20.107 | 29.234 |
| **Male >50 yrs** | 24.959 | 15.831 | 13.049 | 13.092 | 10.107 | 11.247 | 12.227 |

**Table S12.** DRNNAGE validity when utilizing the ensembled randomized network (ERN) to predict age-at-death for a pooled age sample, utilizing the developer’s variable combinations.

| **Traits** | **Accuracy** | **Bias** | **Validity** | **Efficiency** | | |
| --- | --- | --- | --- | --- | --- | --- |
|  | **MAE** |  | **P(α)** | **PIW** | **PIW 95% CI** | |
| **All**  **(m = 64)** | 14.447 | 0.365 | 0.466 | 30.129 | 19.884 |  |
| **Sutures**  **(m = 9)** | 17.032 | 0.758 | 0.822 | 54.839 | 42.053 |  |
| **Axial**  **(m = 12)** | 18.699 | 0.600 | 0.477 | 32.909 | 31.739 |  |
| **Appendicular**  **(m = 23)** | 15.733 | 0.406 | 0.530 | 32.073 | 27.624 |  |
| **Clavicle**  **(m = 2)** | 12.471 | 0.519 | 0.784 | 37.601 | 13.542 |  |
| **1^st^ Rib**  **(m = 2)** | 13.708 | 0.534 | 0.874 | 42.913 | 20.509 |  |
| **Pubic symphysis**  **(m = 3)** | 14.831 | 0.649 | 0.850 | 47.729 | 37.932 |  |
| **Sacroiliac complex**  **(m = 6)** | 16.749 | 0.627 | 0.615 | 40.585 | 30.659 |  |
| **Acetabulum**  **(m = 3)** | 11.984 | 0.546 | 0.798 | 36.834 | 31.099 |  |
| **Standard traits**  **(m = 16)** | 12.288 | 0.414 | 0.639 | 33.426 | 19.752 |  |

**Figure S1.** DRNNAGE validity when utilizing the ensembled randomized network (ERN) to predict age-at-death for decade based segmented age groups, based on the developer’s variable combinations. The number of variables included in each combination is reported as “*m*”, while the number of individuals per age group is given at the top of each bar.


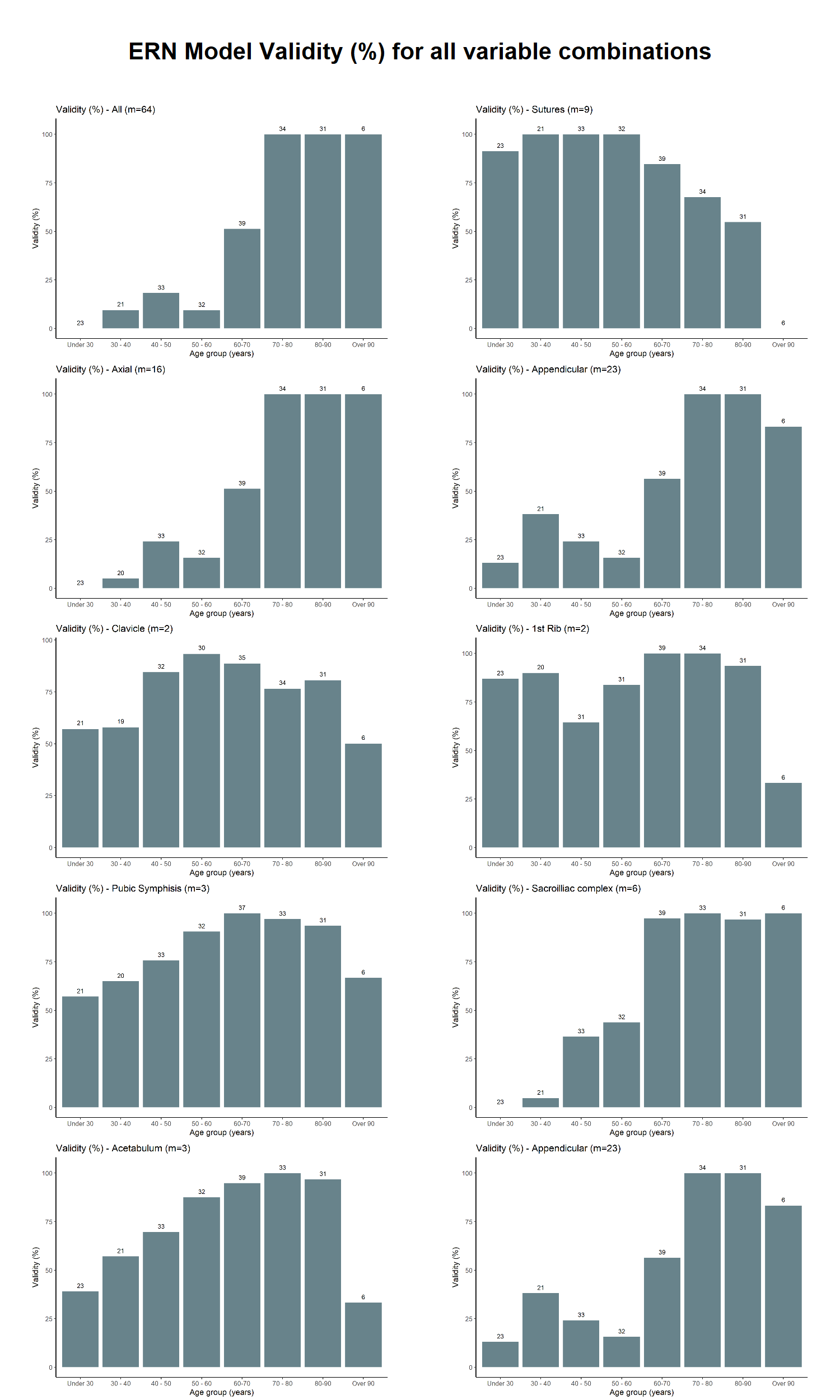

Supplement: Supplementary file 1 — (DOC 370 kb) [file 414_2023_3129_MOESM1_ESM.doc]
